# Supplementary material for: A signal-like role for floral humidity in a nocturnal pollination system
Source: Nat Commun. 2022 Dec 15;13:7773. doi: 10.1038/s41467-022-35353-8 (PMC9755274; doi:10.1038/s41467-022-35353-8)
Supplement: Supplementary file 1 — Supplementary Information [file 41467_2022_35353_MOESM1_ESM.pdf]

Supplementary material

Manuscript: A signal-like role for floral humidity in a nocturnal pollination system

Ajinkya Dahake[1*], Piyush Jain[2], Caleb Vogt[1], William Kandalaft[1], Abraham Stroock[3], Robert A. Raguso[1]

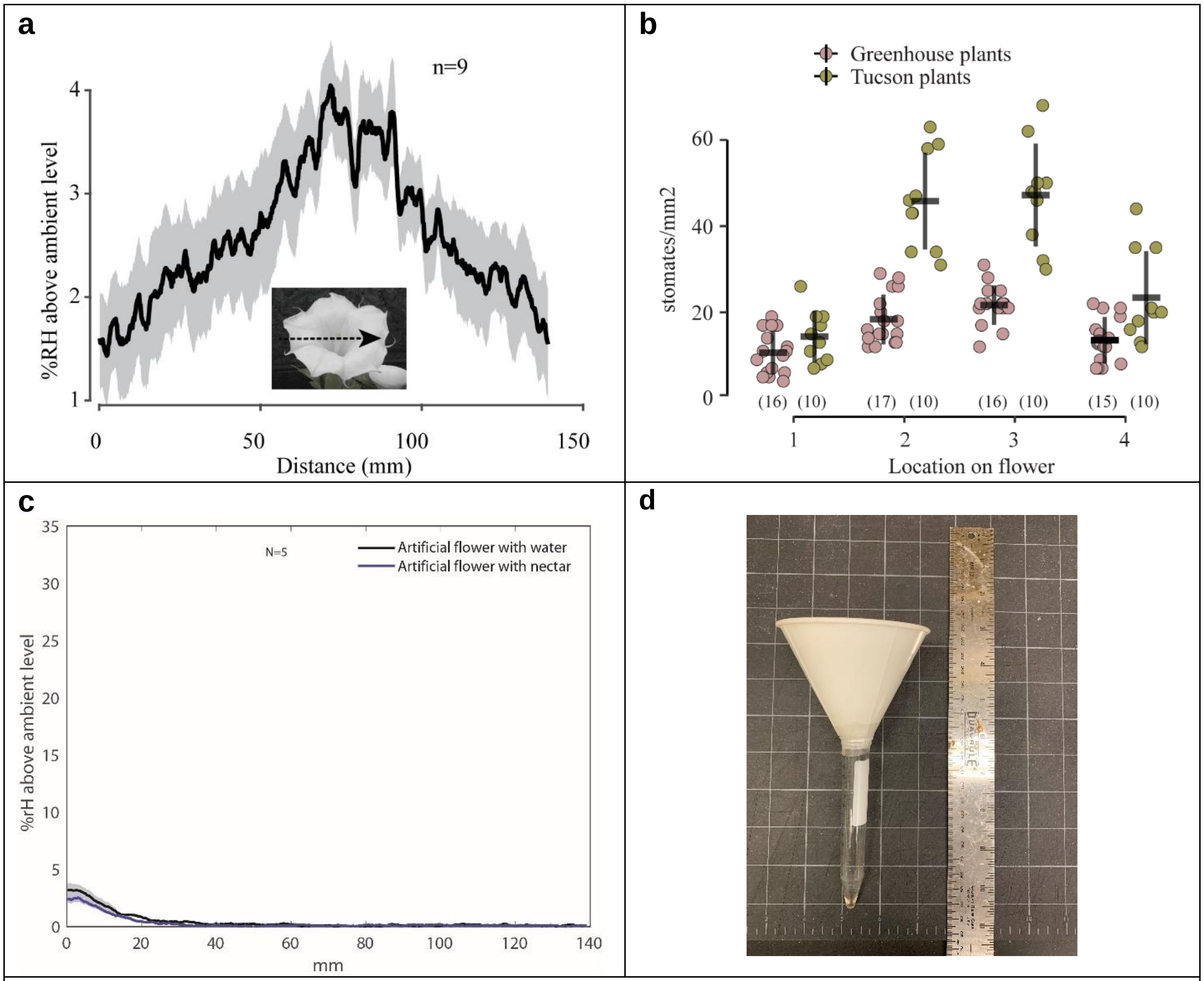

Supplementary Fig. 1. a Floral humidity measured along a horizontal transect 0.5 cm above the flower opening. The inset image of a *Datura wrightii* flower with the arrow shows a typical transect trajectory. The black solid line shows the mean and the shaded area (gray) shows the SEM. The sample size is indicated in the top-right corner. **b** Stomatal counts across 4 locations, same as Fig. 1e in the manuscript, but on the outer surface of the flowers (abaxial) from greenhouse-grown plants (magenta) and flowers collected from Tucson, AZ, USA (olive green). Dot plots show stomatal counts of individual flowers. Black lines show the mean (horizontal) ± SD (vertical). Sample sizes are indicated in parentheses. **c** Floral humidity of the artificial flower (as shown in d) with either 200 µl of *Datura* nectar or water in the nectar tube if it were an inadvertent cue for nectar presence in this system as shown previously for *Oenothera cespitosa*[1]. Solid lines show the mean of n=5 transects, and the shaded region (gray) shows the SEM **d** An artificial flower constructed from a 15 ml falcon tube and a 10 cm diameter plastic funnel. Flower dimensions roughly match that of authentic *Datura* flowers. Source data are provided as a Source Data file.

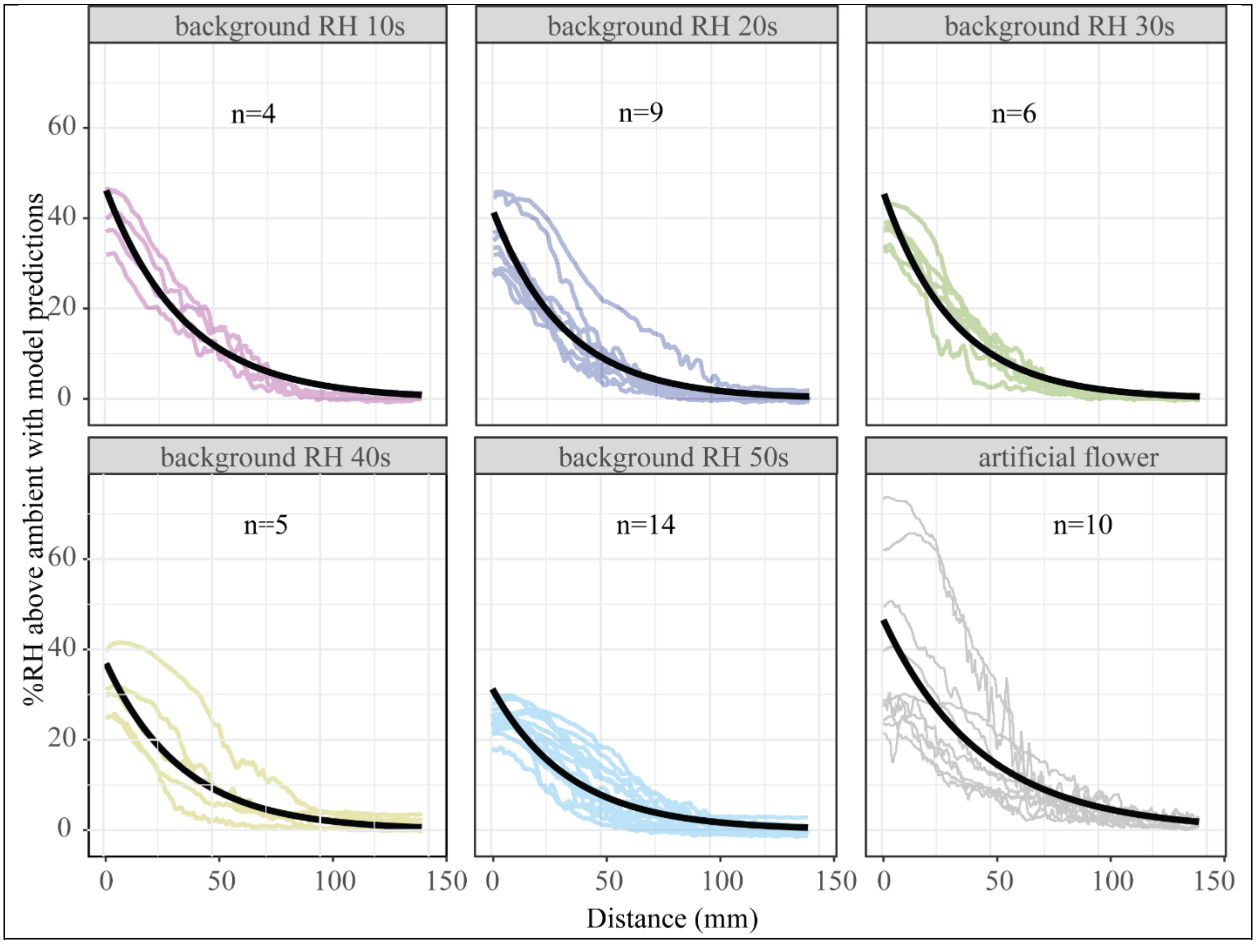

Supplementary Fig. 2 Predicted model fits (solid black line) for floral humidity at different levels of background humidity including the predicted fit for the humid artificial flower used in the behavioral experiments. Individual flower transects are shown for the indicated level of background humidity. Sample sizes are mentioned in each panel.

Supplementary Table 1. Multiple comparisons using pairwise *t*-tests with post hoc Tukey adjustments to the *p*-values of the model estimates for the **decay rate *α*** across the different levels of background humidity as shown in Supplementary Fig. 2

comparisons	estimate	SE	df	T ratio	P-value
teens - twenties	-0.00303879	0.00419275	24653	-0.72	0.95
teens - thirties	-0.00353883	0.00450340	24653	-0.79	0.93
teens - forties	-0.00102431	0.00468254	24653	-0.22	1.00
teens - fifties	-0.00048512	0.00395634	24653	-0.12	1.00
twenties - thirties	-0.00050004	0.00367897	24653	-0.14	1.00
twenties - forties	0.00201448	0.00389620	24653	0.52	0.99
twenties - fifties	0.00255367	0.00298443	24653	0.86	0.91
thirties - forties	0.00251451	0.00422870	24653	0.59	0.98
thirties - fifties	0.00305371	0.00340710	24653	0.90	0.90
forties - fifties	0.00053919	0.00364059	24653	0.15	1.00

Supplementary Table 2. Multiple comparisons using pairwise *t*-tests with post hoc Tukey adjustments to the *p*-values for the **intercept *y0*** across the different levels of background humidity as shown in Supplementary Fig. 2

comparisons	estimate	SE	df	T ratio	P-value
teens - twenties	4.89352959	4.04871812	24653	1.209	0.746
teens - thirties	0.75496044	4.34911691	24653	0.174	1.000
teens - forties	9.30934183	4.51954395	24653	2.060	0.238
teens - fifties	14.9876402	3.81964569	24653	3.924	0.001
twenties - thirties	-4.13856915	3.55136021	24653	-1.165	0.771
twenties - forties	4.41581224	3.75814036	24653	1.175	0.766
twenties - fifties	10.0941106	2.878721	24653	3.506	0.004
thirties - forties	8.55438139	4.07999001	24653	2.097	0.221
thirties - fifties	14.2326798	3.287816	24653	4.329	0.0001
forties - fifties	5.6782984	3.5101558	24653	1.618	0.486

Supplementary Table 3. Floral humidity of *Datura* flowers was measured in their natural habitat during Aug 14-19, 2019, at three locations in Pima county, Tucson, Arizona.

location	Ambient conditions		%ΔRH (mean ± SD)		n	Weather conditions
	% RH	Temp ºC	tube	opening		
University of AZ, experimental plot	29	31.8	27.79±5.63	4.00±4.84	21	breezy, moths probing flowers
Windy Point	24	25.3	29.73±7.29	1.07±0.73	7	windy, no moths
SRER, grassland	33.9	28.7	21.33±5.62	0.69±1.13	9	Overcast and strong gusts of wind, storm approaching.

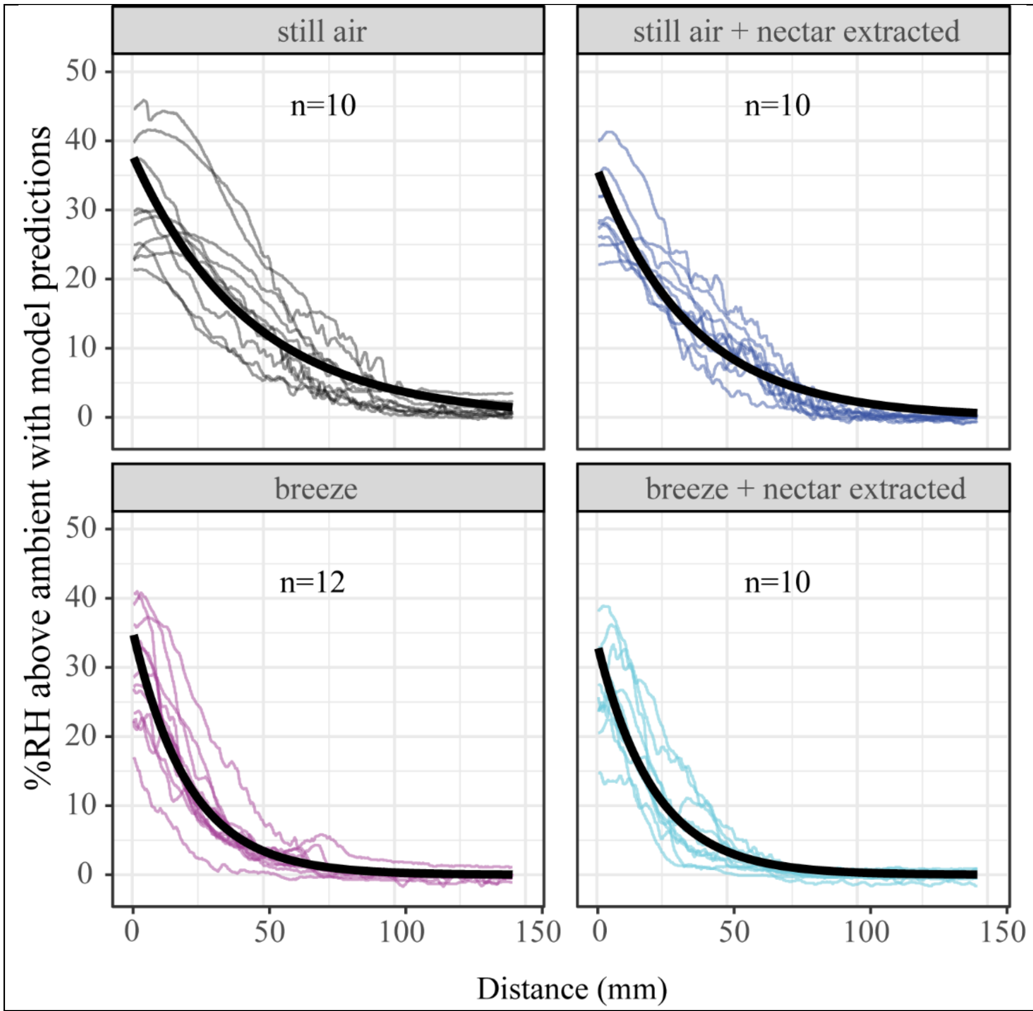

Supplementary Fig. 3 Model predictions (solid black line) for floral humidity were examined under different treatments. Individual flower transects (color-coded) are shown for the indicated treatment. Sample sizes are mentioned in each panel.

Supplementary Table 4 Multiple comparisons using pairwise *t*-tests with post hoc Tukey adjustments to the *p*-values for the **decay rate *α*** across the different floral manipulations as shown in Supplementary Fig. 3

comparisons	estimate	SE	df	T ratio	P-value
Still air – (still air + nectar extracted)	-0.0056754	0.00397353	27251	-1.4283	0.4815598884
Still air - breeze	-0.02521763	0.00381531	27251	-6.6096	0.0000000002
Still air – (breeze+nectar extracted)	-0.02489692	0.00398317	27251	-6.2505	0.0000000025
(Still air + nectar extracted) - breeze	-0.01954223	0.0038161	27251	-5.1210	0.0000018152
(Still air + nectar extracted) - (breeze + nectar extracted)	-0.01922152	0.00398393	27251	-4.8248	0.0000083445
breeze - (breeze + nectar extracted)	0.00032071	0.00382614	27251	0.0838	0.9997890394

Supplementary Table 5. Multiple comparisons using pairwise *t*-tests with post hoc Tukey adjustments to the *p*-values for the **intercept *y0*** across the different floral manipulations as shown in Supplementary Fig. 3

comparisons	estimate	SE	df	T ratio	P-value
Still air – (still air + nectar extracted)	2.04763868	3.853107	27251	0.531	0.951
Still air - breeze	2.67904836	3.68976469	27251	0.726	0.887
Still air - (breeze + nectar extracted)	4.63361166	3.85392027	27251	1.202	0.625
(Still air + nectar extracted) - breeze	0.63140968	3.69000085	27251	0.171	0.998
(Still air + nectar extracted) - (breeze + nectar extracted)	2.58597298	3.85414637	27251	0.671	0.908
breeze - (breeze + nectar extracted)	1.9545633	3.69085005	27251	0.530	0.952

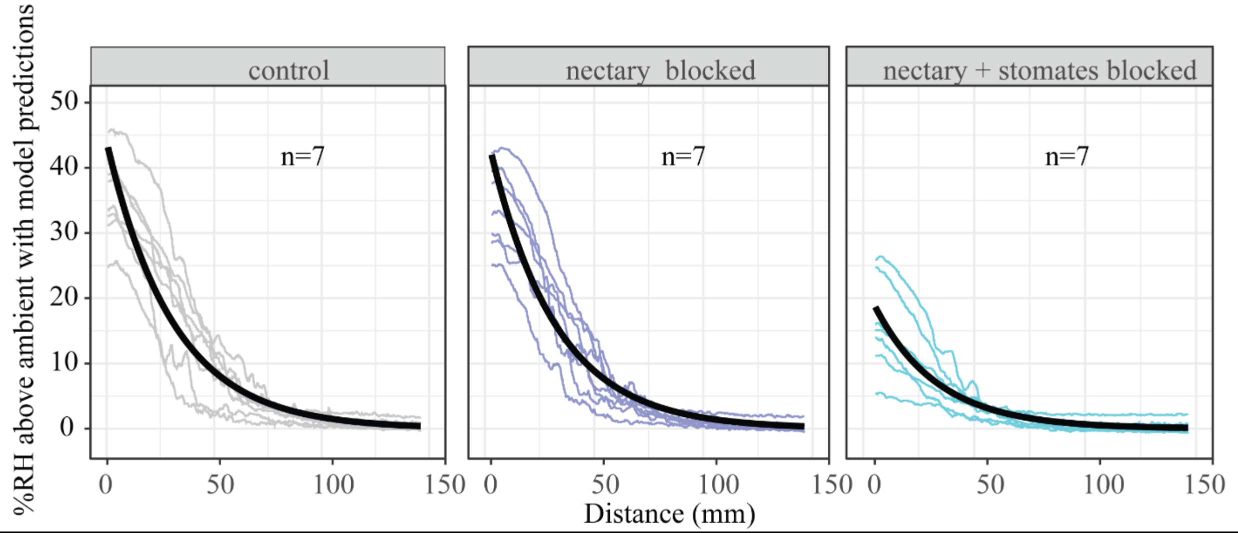

Supplementary Fig. 4 Model predictions (solid black line) for floral humidity of control and manipulated flowers. Individual flower transects for each treatment (color-coded) are shown for the indicated treatment. Sample sizes are mentioned in each panel.

Supplementary Table 6. Multiple comparisons using pairwise *t*-tests with post hoc Tukey adjustments to the *p*-values for the **decay rate *α*** across the different treatments are shown in Supplementary Fig. 4

comparisons	estimate	SE	df	T ratio	P-value
control - nectary blocked	-0.00039462	0.00373999	13624	-0.106	0.994
control - (nectary + stomates blocked)	-0.00195809	0.00376801	13624	-0.520	0.862
nectary blocked - (nectary + stomates blocked)	-0.00156347	0.00376792	13624	-0.415	0.909

Supplementary Table 7. Multiple comparisons using pairwise *t*-tests with post hoc Tukey adjustments to the *p*-values for the **intercept *y0*** across the different treatments are shown in Supplementary Fig. 4

comparisons	estimate	SE	df	T ratio	P-value
control - nectary blocked	1.14949123	4.40171314	13624	0.261	0.96310419
control - (nectary + stomates blocked)	24.6623467	4.40178785	13624	5.603	0.00000007

nectary blocked - (nectary + stomates blocked)	23.5128555	4.40180713	13624	5.342	0.00000028

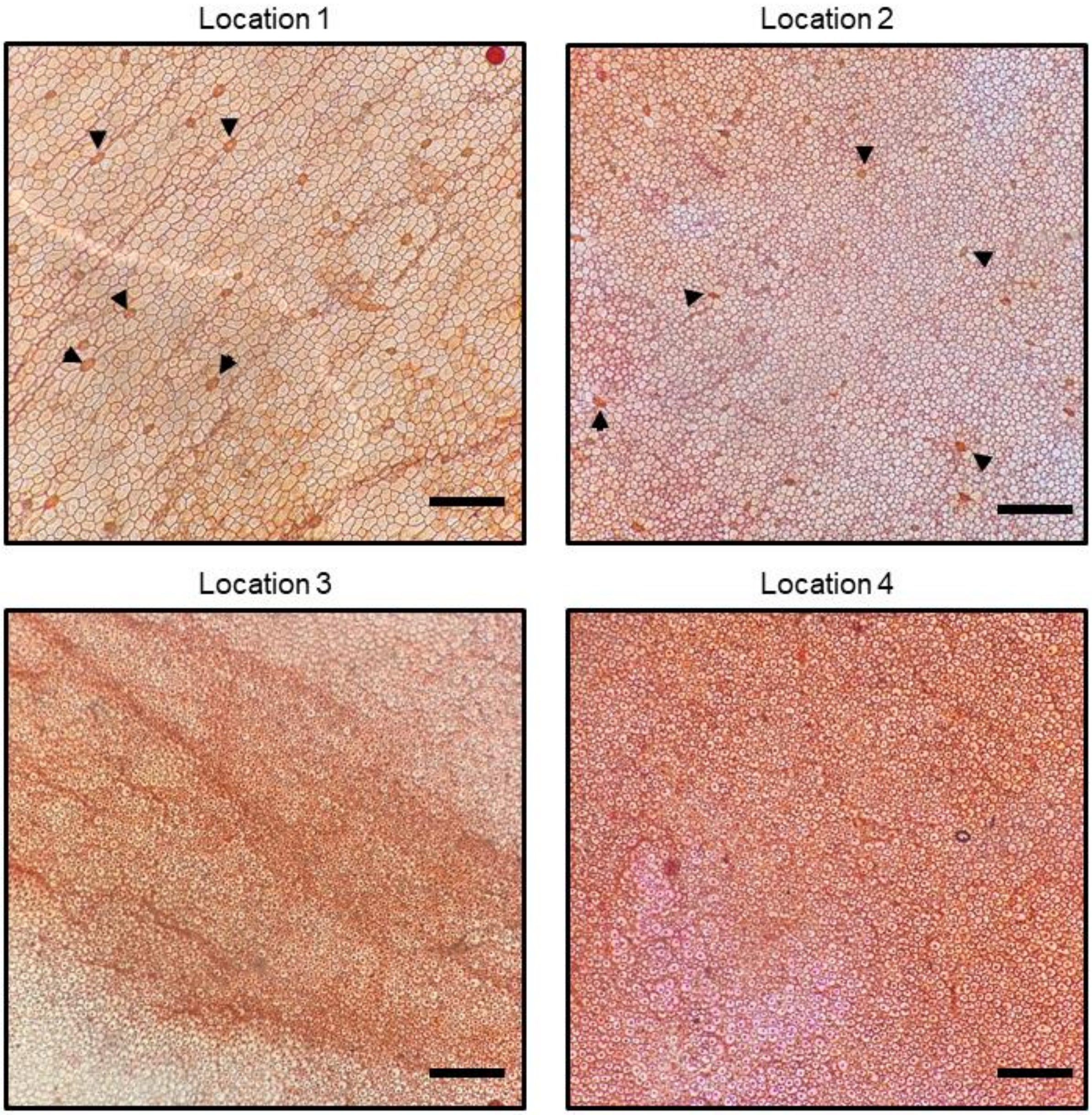

Supplementary Fig. 5 Representative microscope images of the safranin-stained flower peels from the inner surface of the flower (locations depicted in Figure 1e of the manuscript). Arrowheads point towards some of the stomates. No stomates were visible on Locations 3 and 4. Scale bar: 200 µm.

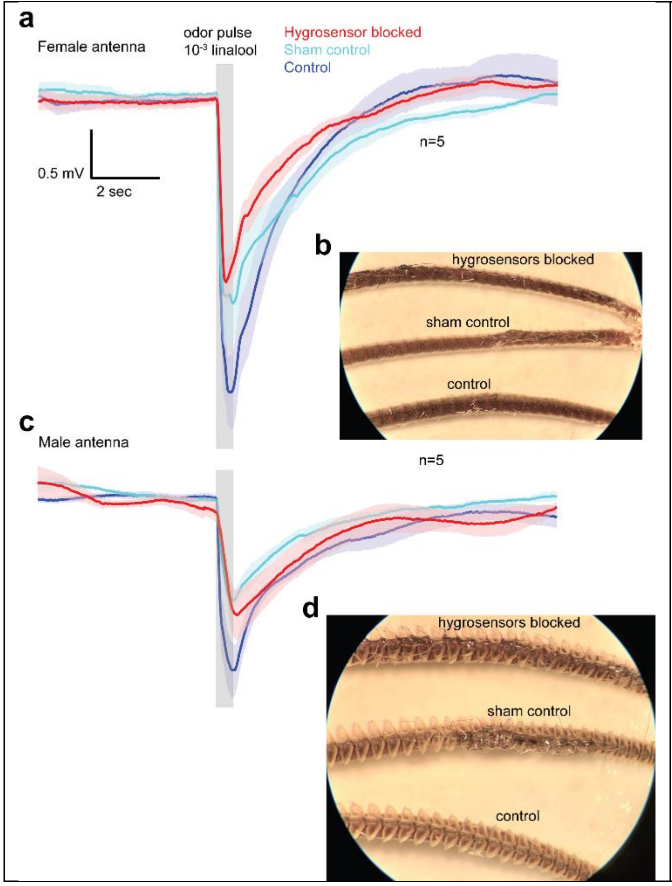

Supplementary Fig. 6 EAG responses of *M. sexta* for the indicated treatments to a 500ms odor pulse of 10^{-3} linalool.

a. Color-coded mean (solid lines) ± SEM (shaded area) responses of n=5 female antennae for each treatment. The scale bar shows the amplitude and the duration of the response.
b. Representative images of the female antenna for each treatment.
c. Color-coded mean (solid lines) and SEM (shaded area) responses of n=5 male antennae for each treatment. Scale bar as above.
d. Representative images of the male antenna for each treatment

Source data are provided as a Source Data file

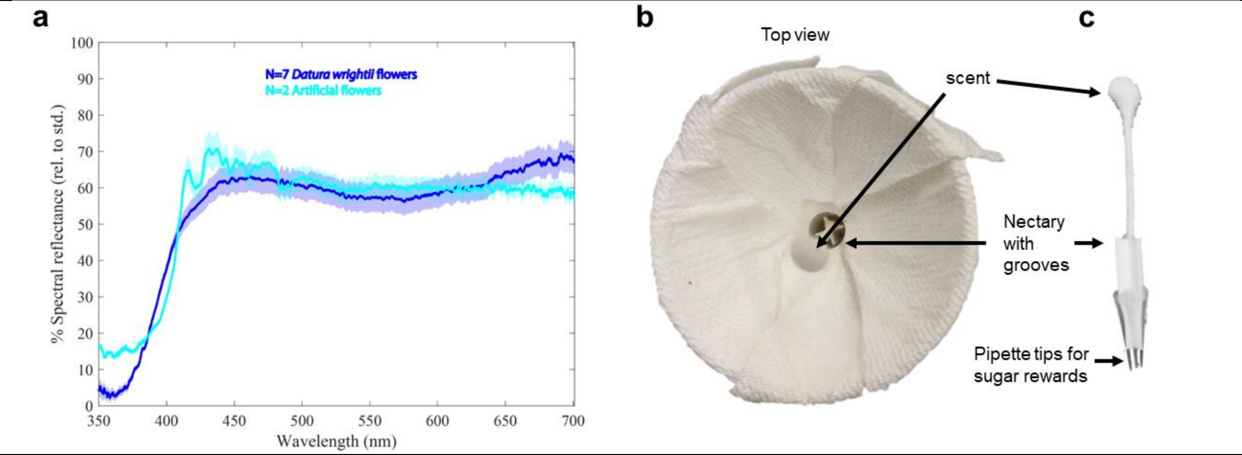

Supplementary Fig. 7 Design of the artificial flower used in the binary choice experiments.

a. Spectral reflectance of n=7 *Datura wrightii* flowers and that of the artificial flower n=2 match closely with each other. Solid lines show the mean, and the shaded area shows the SEM.
b. Top view of the artificial flower showing the four nectary grooves and the Q-tip occupying the space of style and stamens.
c. The internal assembly of the flower tube with the grooves ending in 4 pipette tips holding up to 50 µl sugar solution each. 5 µl Bergamot oil was added to the Q-tips at the start of each night of the experiment.

Source data are provided as a Source Data file.

a

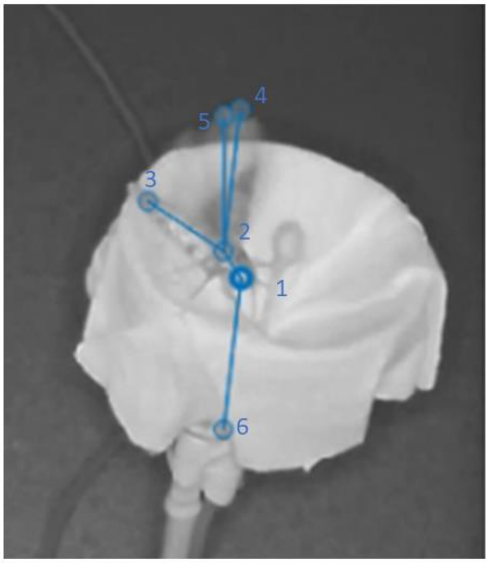

Labeled Nodes

1. **Eye**
2. **Thorax-abdomen bridge**
3. **Abdomen tip**
4. **Left wing**
5. **Right wing**
6. **Proboscis tip**

b

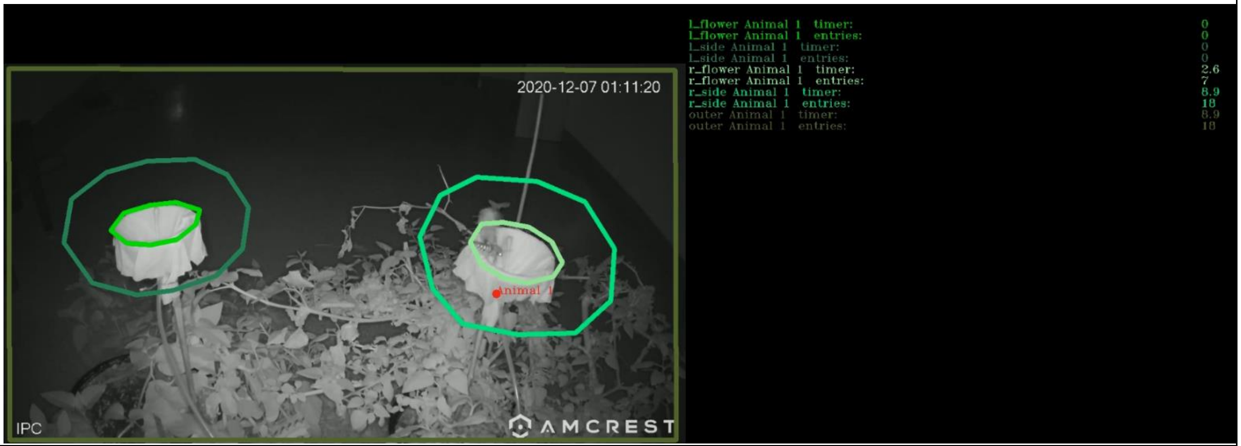

Supplementary Fig. 8 Methods for Behavior video analysis

a. Moth body parts labeled using SLEAP v1.1.5, a deep-learning-based framework for estimating animal body part positions[2,3]
b. Behavioral analysis of the SLEAP labeled moth body parts by tracking their frequency and time spent in the drawn region of interest (green circles) around flowers using SimBA, an open-source toolkit for behavioral analysis[4]

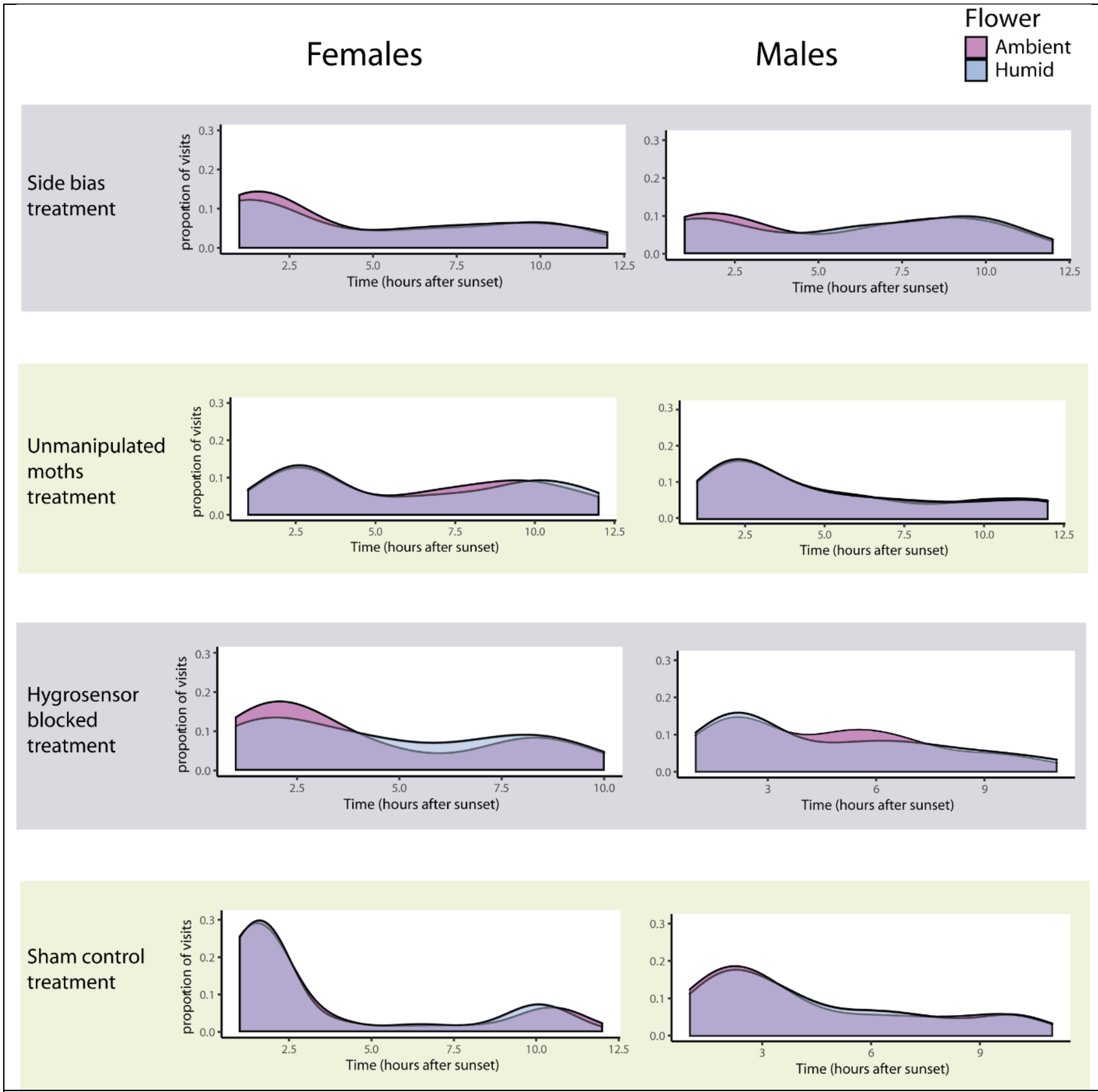

Supplementary Fig. 9 Density plots show the proportion of visits by male and female moths by each hour of the night. A visit is defined as the appearance of the moth body part within the region of interest shown in Fig. 4c of the main manuscript. Plots are separated by the treatments shown in Figure 4 of the main manuscript which summarizes the metadata. Two important points to note here 1) moths visit both flowers throughout the night, 2) Peak activity is within the first 2-5 hours after sunset, followed by a dip around midnight, and increases slightly again in the last 2-3 hours before sunrise. Yellow background indicates the treatments where moths show a significant preference for probing duration and the number of entries in the humid flower, whereas the gray background reflects the treatments where moths showed no preference for either flower. Source data are provided as a Source Data file.

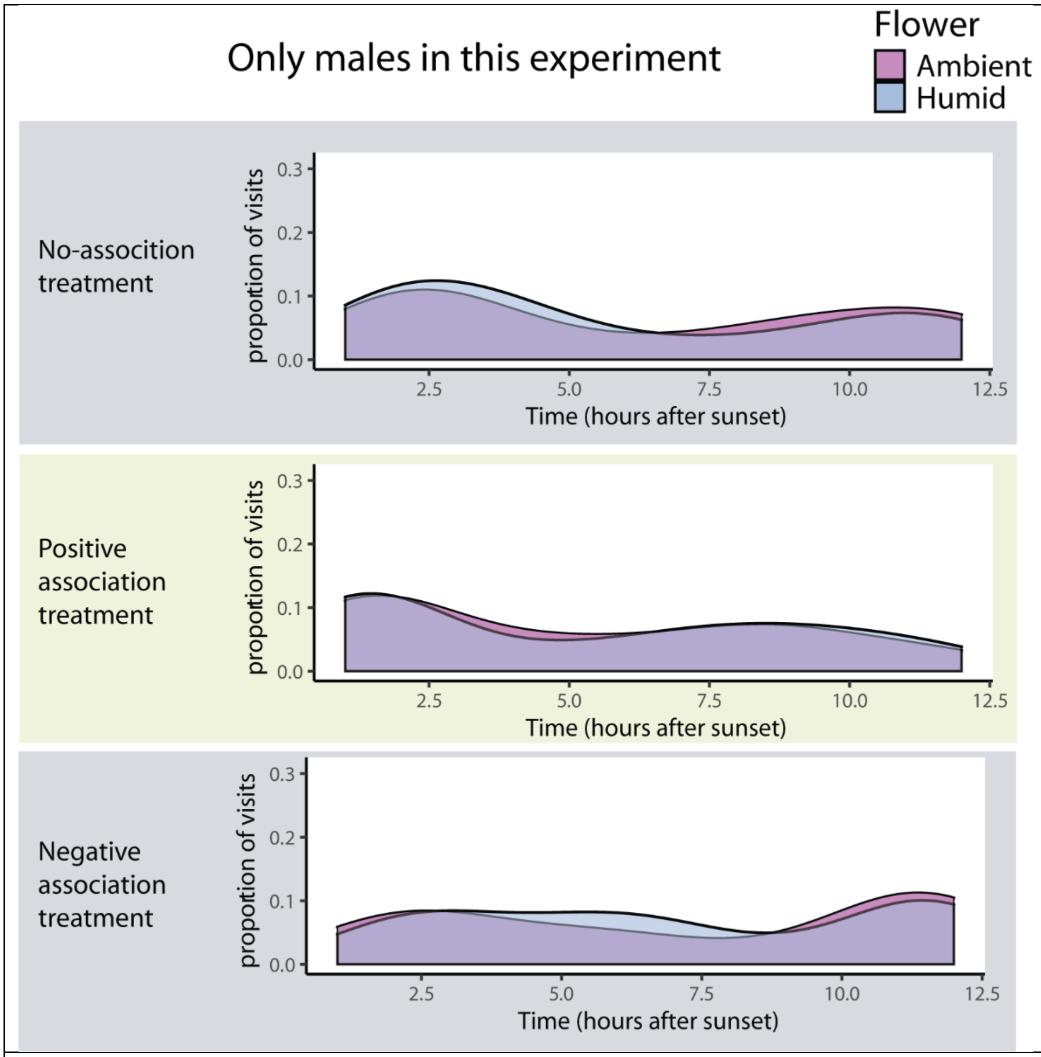

Supplementary Fig. 10 Density plots show the proportion of visits by male moths by each hour of the night. A visit is defined as the appearance of the moth body part within the region of interest shown in Fig. 4c of the main manuscript. Plots are separated by the treatments shown in Figure (5d-i) of the main manuscript which summarizes the metadata. As in the previous figure 1) moths visit both flowers throughout the night, 2) Peak activity is within the first 2-5 hours after sunset, followed by a dip around midnight, and goes up slightly again in the last 2-3 hours before sunrise. Yellow background indicates the treatment where moths show a significant preference for probing and the number of entries in the humid flower and supports the prediction for both hypotheses H_1 and H_2 outlined in table 1 of the main manuscript. The gray background reflects the treatments where neither hypothesis was completely supported. Source data are provided as a Source Data file.

Supplementary Table 8. Data showing the surface area and volume of Datura flowers.

Flower #	Surface area (cm^2)	Flower #	Volume (cm^3)
1	154.03	1	60
2	173.39	2	56
3	172.69	3	80
4	159.17	4	91
5	155.41	5	80

6	165.32	6	90
7	147.78	7	90
8	188.14	8	97.5
		9	100
		10	95
**Mean**	**164.49**	**Mean**	**83.95**
**SD**	**12.28**	**SD**	**14.40**

Supplementary Table 9. Data showing the water budget of a *Datura* flower. Raw values for flower and nectar mass and the percent water mass allocation to flower vs. nectar.

flower #	fresh flower weight (g)	dry flower weight (g)	% Floral water mass	Fresh nectar weight (g)	% Nectar water mass	% Total floral water mass
1	6.33	1.02	83.89	0.16	2.53	86.41
2	6.68	1.16	82.63	0.13	1.95	84.58
3	5.85	0.84	85.64	0.14	2.39	88.03
4	5.23	0.66	87.38	0.11	2.10	89.48
5	6.31	0.86	86.37	0.09	1.43	87.80
6	6.71	0.98	85.39	0.18	2.68	88.08
**mean**	**6.19**	**0.92**	**85.22**	**0.14**	**2.18**	**87.40**
**SD**	**0.51**	**0.16**	**1.56**	**0.03**	**0.42**	**1.54**

Supplementary References

1 von Arx, M., Goyret, J., Davidowitz, G. & Raguso, R. A. Floral humidity as a reliable sensory cue for profitability assessment by nectar-foraging hawkmoths. *Proc Natl Acad Sci U S A* **109**, 9471-9476, doi:10.1073/pnas.1121624109 (2012).

2 Pereira, T. D. *et al.* Fast animal pose estimation using deep neural networks. *Nat. Methods* **16**, 117-125, doi:10.1038/s41592-018-0234-5 (2019).

3 Pereira, T. D. *et al.* SLEAP: A deep learning system for multi-animal pose tracking. *Nat. Methods*, doi:10.1038/s41592-022-01426-1 (2022).

4 Nilsson, S. R. *et al.* Simple Behavioral Analysis (SimBA) – an open source toolkit for computer classification of complex social behaviors in experimental animals. *bioRxiv*, doi:10.1101/2020.04.19.049452 (2020).
