# Supplementary material for: A signal-like role for floral humidity in a nocturnal pollination system
Source: Nat Commun. 2022 Dec 15;13:7773. doi: 10.1038/s41467-022-35353-8 (PMC9755274; doi:10.1038/s41467-022-35353-8)
Supplement: Supplementary file 3 — Description of Additional Supplementary Files [file 41467_2022_35353_MOESM3_ESM.pdf]

Description of Additional Supplementary Files

File Name: Supplementary Movie 1
Description: Shows a 3x sped-up video of a *Manduca sexta* moth interacting with a *Datura wrightii* flower with simultaneous measurements of the floral humidity of the flower. This video is in reference to figure 1f in the manuscript.
