# Supplementary material for: A signal-like role for floral humidity in a nocturnal pollination system
Source: Nat Commun. 2022 Dec 15;13:7773. doi: 10.1038/s41467-022-35353-8 (PMC9755274; doi:10.1038/s41467-022-35353-8)
Supplement: Supplementary file 5 — Reporting Summary [file 41467_2022_35353_MOESM5_ESM.pdf]

nature portfolio

Corresponding author(s): Ajinkya Dahake
asd244@cornell.edu

Last updated by author(s): Nov 15, 2022

Reporting Summary

Nature Portfolio wishes to improve the reproducibility of the work that we publish. This form provides structure for consistency and transparency in reporting. For further information on Nature Portfolio policies, see our Editorial Policies and the Editorial Policy Checklist.

Statistics

For all statistical analyses, confirm that the following items are present in the figure legend, table legend, main text, or Methods section.

n/a	Confirmed	
☐	☒	The exact sample size (*n*) for each experimental group/condition, given as a discrete number and unit of measurement
☐	☒	A statement on whether measurements were taken from distinct samples or whether the same sample was measured repeatedly
☐	☒	The statistical test(s) used AND whether they are one- or two-sided<br>*Only common tests should be described solely by name; describe more complex techniques in the Methods section.*
☒	☐	A description of all covariates tested
☐	☒	A description of any assumptions or corrections, such as tests of normality and adjustment for multiple comparisons
☐	☒	A full description of the statistical parameters including central tendency (e.g. means) or other basic estimates (e.g. regression coefficient) AND variation (e.g. standard deviation) or associated estimates of uncertainty (e.g. confidence intervals)
☐	☒	For null hypothesis testing, the test statistic (e.g. *F*, *t*, *r*) with confidence intervals, effect sizes, degrees of freedom and *P* value noted<br>*Give P values as exact values whenever suitable.*
☒	☐	For Bayesian analysis, information on the choice of priors and Markov chain Monte Carlo settings
☒	☐	For hierarchical and complex designs, identification of the appropriate level for tests and full reporting of outcomes
☒	☐	Estimates of effect sizes (e.g. Cohen's *d*, Pearson's *r*), indicating how they were calculated

Our web collection on statistics for biologists contains articles on many of the points above.

Software and code

Policy information about availability of computer code

Data collection	Open-source softwares: SpikeHound v1.2 was used to collect neural spike data.<br>All other data were collected in Microsoft Excel version 2210
Data analysis	Commercial softwares: MATLAB R2019a<br>Open-source softwares: R v4.1.1, SLEAP v1.1.5, SimBA v1.3, Arduino IDE, Waveclus 3.0, SpikeHound v1.2<br>Custom code: custom codes were written in MATLAB R2019a to operate the stimulus delivery setup, to align the stimulus with the neural spike data, and to allign moth flower probing video with floral humidity.<br>https://github.com/DahakeAjinkya/Floral-humidity-matlab-files.git

For manuscripts utilizing custom algorithms or software that are central to the research but not yet described in published literature, software must be made available to editors and reviewers. We strongly encourage code deposition in a community repository (e.g. GitHub). See the Nature Portfolio guidelines for submitting code & software for further information.

Data

Policy information about availability of data

All manuscripts must include a data availability statement. This statement should provide the following information, where applicable:
- Accession codes, unique identifiers, or web links for publicly available datasets
- A description of any restrictions on data availability
- For clinical datasets or third party data, please ensure that the statement adheres to our policy

The datasets generated during and/or analysed during the current study have been as a Source Data file and within the Supplementary data file

Field-specific reporting

Please select the one below that is the best fit for your research. If you are not sure, read the appropriate sections before making your selection.

☐ Life sciences ☐ Behavioural & social sciences ☒ Ecological, evolutionary & environmental sciences

For a reference copy of the document with all sections, see nature.com/documents/nr-reporting-summary-flat.pdf

Ecological, evolutionary & environmental sciences study design

All studies must disclose on these points even when the disclosure is negative.

Study description	To evaluate the effect of nectar and breeze on floral humidity the treatments were as follows: still air, breeze, nectar extracted, nectar extracted+breeze. These treatments were performed sequentially on 10 flowers from different greenhouse-grown plants.<br>To evaluate the effect of nectary and stomatal blockage, 7 flowers were used from different greenhouse-grown plants.<br>For the moth behavior experiments, the treatments were: control, hygrosensor blocked, and sham control. Sample sizes ranged between 7-12 nights with 2-4 moths each night. The number of videos/night varied between nights.
Research sample	The research sample constitutes the Datura wrightii plant (Nightshade family) and its hawkmoth pollinator, Manduca sexta (Tobacco hornworm moth). This research sample was chosen because of the prior natural history and ecological data available on this insect-plant mutualism. M. sexta was raised in the lab at Cornell University from a parent population from Arizona, Tucson, USA.<br>For the innate behavioral response experiment, both male and female moths were used. We excluded the females from the behavior experiments that included nectar rewards in the flowers (Fig.5) to avoid the oviposition context. In all experiments, moths were 3-5 days old, starved, and flower-naive. The manipulations on flowers include extraction of nectar, blocking off the nectaries, and inner side stomates with petroleum jelly. The manipulations to moths include occluding the leading edge of their entire antennae with UV hardened glue to block the hygrosensors, whereas for the sham control only 5-10 segments of the antennae were occluded leaving rest of the antennae intact.
Sampling strategy	No prior sample size calculation was performed. Sample sizes were based on the availability of flowers and moths to work with. The motion-sensing camera ensured all interactions between moths and the artificial flowers were captured. This setup generated sufficient data to perform robust statistical analysis.
Data collection	Ajinkya Dahake collected all the data in this manuscript. Floral humidity curves, stomatal counts, and moth behavior experiments were conducted in Prof. Robert Raguso's laboratory at Corson Mudd Hall, Cornell University, Ithaca, NY, USA. The humidity data on the wild plants were collected at three different sites around Tucson, Arizona, USA. Details are provided in the Methods section of the manuscript and the supplementary information.
Timing and spatial scale	Floral humidity transects were taken in the laboratory throughout the year from 2018 to 2020 as per availability of the flowers. An entire year of floral humidity sampling ensured data collection in a range of background humidity from 10%RH to 60%RH. Field sampling of floral humidity of naturally growing Datura plants in Tucson, Arizona, USA occurred in Aug 2019 and July 2021. Moth behavior experiments were conducted in the laboratory from February 2020 to January 2021.
Data exclusions	A few moth behavior videos were excluded from further analysis. This has been mentioned in the Methods section (details below).<br>For the "hygrosensor blocked" treatment, moth antennae were checked under a microscope the following morning after the experiment to ensure the glue was intact. If a significant portion of the glue was missing from the antennae, videos from such nights were excluded from further analysis. This occurred for 2 nights out of the 12 nights for males and 3 nights out of 13 for females, effectively resulting in a sample size of n=10 nights for each sex.
Reproducibility	Sample sizes are mentioned for each panel either in the figure legends or directly on the figures in parenthesis or in the form "n=12". All attempts to repeat the experiments were succesful.
Randomization	The allocation of flowers and moths into different treatments was random.
Blinding	Blinding was not possible for data acquisition, but for the moth behavior video analysis, we used an unbiased approach by tracking moth body parts using a body pose-estimation open-source software SLEAP v1.1.5 and mapping the body part coordinates in the 2D space around the artificial flowers using the open-source software SimBA v1.3 developed by the Golden Lab.

Did the study involve field work? ☒ Yes ☐ No

Field work, collection and transport

Field conditions	This data is available in the supplemetary material (Table S3).
Location	This data is provided in the manuscript in the methods section.<br>University of Arizona experimental plot at Roger Road (32°16'41.3"N 110°56'18.5"W, 715m)<br>Windy point in the Santa Catalina mountains (32°22'07.0"N 110°43'00.8"W, 2013m)<br>Santa Rita Experimental Range grasslands (31°47'01.5"N 110°49'32.3"W, 1322m).
Access & import/export	Datura wrightii seeds that belonged to the population from Tucson, Arizona, USA, were requested from the seed bank at Radboud University, Nijmegen, The Netherlands (Accession number: 944750169). This is mentioned in the Methods section of the manuscript.

Disturbance	The study caused no disturbance to the natural habitat because all data were collected non invasively.

Reporting for specific materials, systems and methods

We require information from authors about some types of materials, experimental systems and methods used in many studies. Here, indicate whether each material, system or method listed is relevant to your study. If you are not sure if a list item applies to your research, read the appropriate section before selecting a response.

Materials & experimental systems

n/a	Involved in the study
☒	☐ Antibodies
☒	☐ Eukaryotic cell lines
☒	☐ Palaeontology and archaeology
☐	☒ Animals and other organisms
☒	☐ Human research participants
☒	☐ Clinical data
☒	☐ Dual use research of concern

Methods

n/a	Involved in the study
☒	☐ ChIP-seq
☒	☐ Flow cytometry
☒	☐ MRI-based neuroimaging

Animals and other organisms

Policy information about studies involving animals; ARRIVE guidelines recommended for reporting animal research

Laboratory animals	Manduca sexta catterpillars were raised in the lab from egg to adult year round on a cornmeal based diet. The lab colony was established from a parent population of moths from Tucson, Arizona, USA. Both males and females were used in performing the experiments. All moths used in the experiments were between 3 to 5 days old.
Wild animals	This study did not involve wild animals.
Field-collected samples	For stomatal counts of the flowers from the naturally growing Datura wrightii population from Tucson, AZ, USA, flowers were stored in a refrigerator inside Ziploc bags with wet paper towels until floral peels were taken.
Ethics oversight	No ethical approval was necessary because the study species is an insect, however, the guidelines provided by Cornell University's Institutional Animal Care and Use Committee (IACUC) were followed to minimize the stress on animals at every stage of their life cycle.

Note that full information on the approval of the study protocol must also be provided in the manuscript.
